# On video lectures during remote teaching and beyond

**DOI:** 10.1007/s00216-022-03983-y

**Published:** 2022-03-19

**Authors:** Gunnar Schwarz, Davide Bleiner, Detlef Günther

**Affiliations:** 1 Laboratory of Inorganic Chemistry, Department of Chemistry and Applied Biosciences, ETH Zurich, Vladimir-Prelog-Weg 1, 8093 Zürich, Switzerland; 2 Laboratory for Advanced Analytical Technologies, Swiss Federal Laboratories for Materials Science & Technology (Empa), Überlandstrasse 129, 8600 Dübendorf, Switzerland; 3 Department of Chemistry, University of Zurich, Winterthurerstrasse 190, 8057 Zürich, Switzerland

## Abstract

**Supplementary Information:**

The online version contains supplementary material available at 10.1007/s00216-022-03983-y.

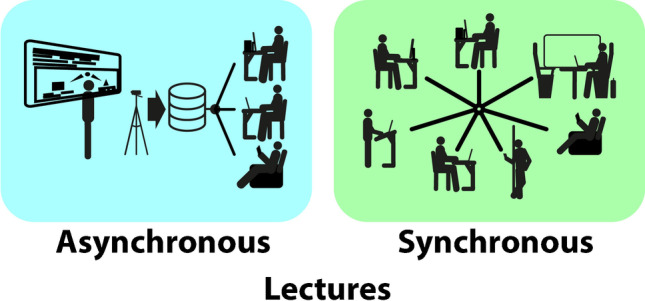

## Introduction

The days of distance education by snail mail are something of the past. The internet enables the rapid transmission and exchange of information across the globe. Online education, digital learning materials and resources generally increase flexibility in terms of time, place and pace, interactivity, variety of learning materials provided, and access. This has encouraged distance learning and also led to the development of massive open online courses (MOOCs) to disseminate lecture and learning materials and keep in touch with students [1–3]. Moreover, digital technologies make the production and distribution of video lectures affordable. In addition to formal education, recordings of lectures are also being used to reach broader and more public audiences (e.g. YaleCourses (https://www.youtube.com/user/YaleCourses), Gresham College (https://www.gresham.ac.uk/).^1^

Online training is a particular form of distance education that has been studied and compared to face-to-face instruction for decades. Given the diversity of approaches and implementations, results have been inconsistent. Overall, meta-analyses such as the study by Bernard et al. [1] have found no overall difference between modalities, but the authors emphasized the wide variance in results. It should be emphasized that it is not the modality per se, but the specific conditions that are responsible for success or failure. With respect to education in chemistry, Cooper and Stowe [4] pointed out that there is little evidence to date that putting any part or a complete lecture course online has significant impact on learning.

In 2020, the COVID-19 pandemic forced instructors around the world into distance learning. While this took place under varying conditions, it was largely achieved online using and adapting material and additional videos, either recorded or livestreamed. Because there was no advance notice, instructors had insufficient time to plan and adjust. It can be assumed that many conversions were made in haste and with an eye toward convenience and “least resistance” [5]. Over the last months, chemistry lecturers presented their various approaches to remote teaching under pandemic circumstances. For example, asynchronous lectures were recorded in lecture halls [6], synchronous videoconference classes were recorded and made available [7], and hybrid courses combined recorded video lectures (e.g. voice-over presentation slides [8, 9]) and live videoconference discussions [7, 9–13].

In short, online education and the use of video, either as a supplement to face-to-face courses, in blended approaches, or in online-only courses, are increasing in today’s universities. The aim of this paper is to provide an overview of the different modalities of lecture delivery, share experiences of delivering asynchronous and synchronous video lectures remotely, and reflect on students’ feedback. In all cases, the goal was to maintain a lecture hall–like experience. We also explore possible future roles of video in supporting lectures. An account of the ongoing debate about the pros and cons of lectures in higher education is beyond the scope of this paper; interested readers are referred to various contributions [14–18]. Nevertheless, this is an occasion to consider, assess, and reflect on opportunities of video lecture formats for chemistry education.

## Lecture video formats

The requirement to move teaching online in early 2020 presented instructors with the fundamental question of whether to offer lectures synchronously or asynchronously. The former typically involves videoconference systems, while the latter relies on pre-recorded lectures that can be accessed via learning management systems such as Moodle, Canvas, or Blackboard, or other cloud services. In terms of differences in pedagogy, synchronous teaching allows for easier two-way live communication. In contrast, asynchronous teaching offers more flexibility due to on-demand availability (Table 1). The different modalities can be combined to mitigate disadvantages, e.g. by recording synchronous lectures.

**Table 1:**
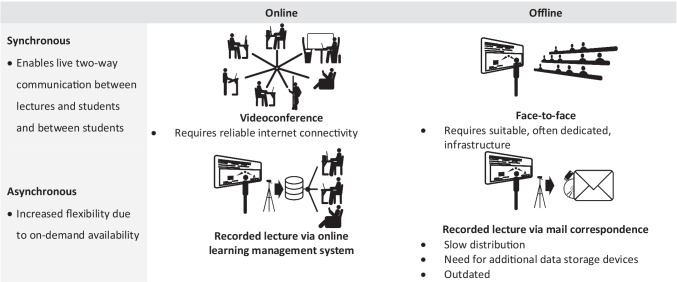
Different lecture modalities, contrasting online with offline and synchronous with asynchronous. Special requirements and selected disadvantages are noted. Combinations of the modalities are possible to mitigate disadvantages. Adapted from and extended upon [19]

**Fig. 1:**
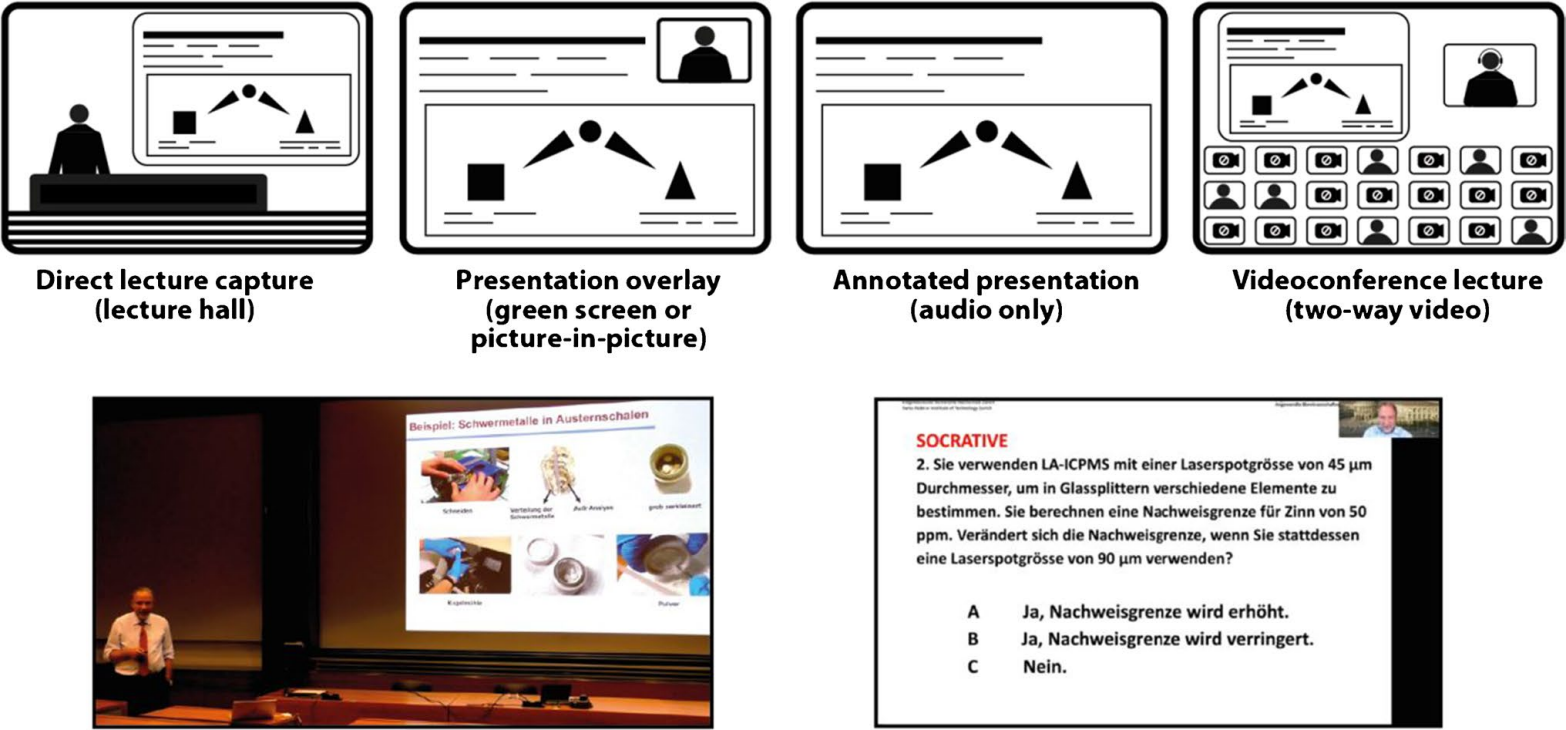
Top: examples for lecture video layouts. Instead of in-advance prepared presentation slides, also on-the-fly handwritten notes can be shown (e.g. chalk or white board). Bottom: Screenshots from a lecture capture in an otherwise empty lecture hall (left) and a live videoconference lecture recording (left)

Lecture videos with accompanying presentation slides can be arranged in a variety of ways (Fig. 1, top) that are largely independent of the delivery mode as a lecture can be recorded in the lecture hall and broadcast asynchronously or streamed live as a videoconference. Chen et al. [20] found that the learning performance of direct lecture recording and picture-in-picture recording was better than that of audio-only recording. The latter resulted in longer sustained attention, but also increased cognitive load. Others [21, 22] emphasized the importance of displaying the instructor, at least during an introduction [8]. Different technical requirements alone may lead lecturers to favour one format over another. With insufficient technical equipment, support, or unreliable internet connectivity [9, 23] asynchronous lectures are preferred [9]. Guo et al. [21] provided general advice for utilizing videos for online courses based on their physics MOOCs. In particular, they suggested that the production of lecture videos should be geared toward a “first-time viewing experience” by polishing the material, e.g. removing unnecessary pauses or filler words (“humm”, “uhhh”). In contrast, tutorial videos usually focus on procedural knowledge and are viewed repeatedly, so they may include chapter markers or text blocks to signify transitions and facilitate skimming of information.

## Course description and lecture modalities

Element Analysis is part of the undergraduate courses Analytical Chemistry I and II, held each year at ETH Zurich, and has already been described in more detail elsewhere [24]. In brief, Element Analysis covers basics in quantitative analysis, such as the analytical process, sampling and sample preparation, and calibration as well as inductively coupled plasma optical emission spectroscopy (ICP-OES), atomic absorption spectroscopy (AAS), X-ray fluorescence spectroscopy (XRF), and inductively coupled plasma mass spectrometry (ICP-MS). The course consists of one 45-min lecture per week and non-mandatory weekly problem sets. As of the institution’s regulations, only attending the final written exam is required; there were no other mandatory or graded activities. Typical enrolment is 180 students (mostly majoring in Chemistry and Chemistry Engineering, Interdisciplinary Sciences, and Material Sciences) for the autumn semester (Analytical Chemistry I) and 120 students for the spring semester (Analytical Chemistry II, with the same majors but not Material Sciences).

After the sudden switch to distance education in March 2020, we planned to provide a “course and lecture experience” as similar as possible to the face-to-face event and considered several options (Fig. 1). Our institution gave free rein about details and technical support to implement different approaches, but required that lectures needed to be recorded in any case for subsequent views by students, who were not able to attend and synchronous meetings. Some institutions suggested asynchronous lectures for courses with high enrolment [6]. Furthermore, as we aimed for a lecture hall–like experience, we decided on a mode similar to recorded lectures for the general public, i.e. recordings in a lecture hall where both the instructor and the presentation slides are visible. This gave us the opportunity to record lectures at more convenient times, e.g. during weekends for some instances, and records multiple lectures in one session. During the planning phase, it became clear from participating in some videoconferences that audio quality is a critical factor in ensuring that students could follow several video lectures over the course of a day. To this end, we used a wireless microphone system for the recordings to capture the instructor’s voice directly.

Therefore, lectures were pre-recorded for asynchronous delivery in spring 2020 in a large lecture hall and made available to the students from the time of the scheduled lectures via a learning management system to the end of the academic year. As with face-to-face lectures, the videos included regular prompts, about three per 45-min lecture, for participation in a classroom response poll system [24, 25] and some open-ended questions. The polls for a lecture were open during the entire day, but only a few responses were received after the scheduled lecture time slot. Because students may have questions or need clarifications, the instructor and lecture assistant were also available via videoconference after each of the scheduled lecture times. In addition, students were encouraged to ask questions or make comments via the classroom response system (anonymous), email, or the learning management system. The recorded video lectures were intentionally kept shorter than 45 min (approximately 30–40 min) to allow students time to engage with the various assignments embedded in the videos and to participate in the voting processes.

The last lecture of the spring semester in 2020 was a summary, another exam preparation exercise and opportunity to ask questions. Since this required live interaction, it was conducted as a videoconference.

Based on positive feedback from our students and reports of technical difficulties with synchronous videoconferencing, we adopted this approach for the new academic year and the autumn 2020 semester. Furthermore, we took the opportunity to deliver the first lecture face to face in the lecture hall (with students present and video recording) for a more authentic and personal introduction, and fully integrated the weekly problem sets into the learning management system.

By the end of 2020, we felt that students had had ample opportunity to experience different lecture formats in their various courses and how they fit into their schedules and study habits under these circumstances. We were willing to change our lecture format to accommodate student preferences, and asked them to participate in an anonymous survey through the learning management system to tell us their preferred lecture format for the spring semester. Although only 18 students participated, possibly indicating that the others had no particular preference, a majority of ten to six chose to switch to synchronous lectures via videoconferencing; two participating students expressed no preference. In part, the low participation rate may also be due to some survey fatigue due to an increased number of requests for feedbacks since the start of remote teaching.

Therefore, lectures during spring 2021 were held synchronously throughout via a videoconferencing system during scheduled lecture times. These lectures were recorded and made available through the learning management system. Further details, including a schedule, can be found in the Supplementary information. All exams were administered in person at the institution. Supplemental regulatory requirements and institution’s policies, such as keeping distance, increased hygiene measures, and mask wearing, were followed at all times.

Throughout, we collected and received anonymous feedback from students via the classroom response system at the end of each lecture, centrally organized course evaluation surveys, and a special survey at the end of the spring 2021 semester asking students about their experiences with synchronous and asynchronous lectures.

## Observations and students’ feedback

After the initial setup and testing of the technical equipment, the additional time spent pre-recording lectures was less than 15 min per lecture, including active work time for video editing. The lack of feedback, often fleeting and vague, from students in the lecture hall to the instructor in the form of facial expressions or subtle behaviour may well be an additional cause for an increase in lecture pace. In fact, the only feedback, and even discrete feedback, that the lecturer received during the recording came from the assistant, who was positioned near the camera to (a) review the recordings and (b) provide the lecturer with a personal visual anchor next to the camera.

In contrast to their usual behaviour in lecture halls, many instructors seemed to sit through videoconference lectures and conference presentations alike. We also did this most of the time because we have adopted this from other videoconference sessions. However, we would like to encourage other lecturers to try putting their devices on an elevated surface, like a larger dresser, so they are able to stand during synchronous video lectures.

Overall, student participation was on par with previous face-to-face sessions for both asynchronous and synchronous lectures. That is, the number of voting participants was similar to that of previous years. At the same time, student questions via email, the learning management platform, the classroom response system, or virtual “office hours” were just as sparse as before. There were a few notable exceptions: The number of students submitting problem sets dropped sharply after the launch of distance learning in spring 2020, when we asked for submissions via email rather than on paper. Subsequently, the percentage of students submitting problem sets via the learning management platform in autumn 2020 increased significantly compared to that of previous years. Finally, for no apparent reason, participation in voting via the classroom response system dropped to only about 20% of lecture videoconference participants in spring 2021. During most of the videoconference lectures, all students had their cameras off, despite the instructor’s explicitly stated request. When asked to do otherwise during the introduction or at the end of the lecture, few students followed this request.

We received praise from students for the first (partial) semester of distance learning (spring 2020) by the university’s course evaluation system with 46 respondents.^2^ Almost 80% of the students agreed or strongly agreed with the statement “The current form of remote works for me”. In autumn 2020, students generally liked the flexibility provided by recordings, but in the centrally organized survey, some students expressed a desire for live videoconference lectures instead of recorded lectures. This prompted us to specifically ask students what lecture format they would prefer for spring 2021, with the above result. Surveys conducted during and at the end of the spring 2021 semester ultimately revealed that a large majority of 80% of survey participants preferred synchronous lectures. Although they valued more flexibility with recorded lectures, students felt that live lectures offered more incentives and allowed for more interaction.

While mean exam scores for Element Analysis in summer 2020, i.e. exams after the course in spring 2020, were at the lower end of the range from previous years, scores in winter and summer 2021 were at the higher end. We do not attribute this solely to online distance learning, as there are many contributing factors, which include but are not limited to the scheduling of the analytical exam within the exam period, change of time students allocate to learn for this course under changing conditions, and whether they enrolled for both or only one of the two courses.

## Discussion

We found many parallels in recent accounts of how lecturers in higher education coped with the sudden shift to online teaching. For example, we agree that it felt “bizarre” [10] to lecture to an empty auditorium, but also note that lecturing via videoconferencing without the ability to perceive the usual audience reactions can be similarly strange. Teaching or technical assistants for synchronous video lectures are certainly very helpful for lecturers to focus on the content and, if necessary, interaction with students [10]. Course preparation time has increased [6, 8, 26], but after initial testing of the equipment, this was less related to the video lectures than to moving the weekly problem sets online as they had to be put into the online form sheet. Although some of the questions were automatically graded, many needed to be graded by hand, which took longer than on paper.

We would like to highlight the following comparison of asynchronous and synchronous video lectures: Our own experience is largely consistent with the feedback from students, who see greater flexibility in the former and the opportunity for live interaction in the latter. Moreover, our students’ feedback is in line with the general observation that students prefer live lectures to recordings [10, 27], because they help them plan their learning [12] and keep up with coursework. However, as medical students themselves noted in a letter to the editor [28], the availability of video recordings can lead to procrastination and an insurmountable workload before exams. Questions or other tasks embedded into the video or follow-up questions may be used to motivate students to keep on schedule. It has been previously emphasized that in distance education, time management and self-efficacy are critical to student success [29, 30]. In contrast, pre-pandemic studies [1] found higher satisfaction and better performance in asynchronous courses, but these studies often involved a larger proportion of part-time students, who are more easily accommodated by the greater flexibility of asynchronous courses.

A number of instructors [7, 11, 13, 31] found videoconference lectures to be less efficient and reported a slower pace than face-to-face courses, especially in relation to interaction with or amongst students. Although this was not particularly evident in our lectures, a largely slower pace of online interaction in synchronous lectures would impose a significant burden when the assigned class time is short, i.e. 45 min per week in our case.

As mentioned earlier, a prominent feature of synchronous lectures is the opportunity for live interaction. Like others [6, 10, 12, 13, 32], we employed quizzes with feedback from the classroom and engaged students via a learning management system for active learning components, which is beneficial for courses with many participants [24]. These can be used for both asynchronous and synchronous lectures. Distinct active learning components during synchronous lectures such as collaborative groups or a flipped classroom approach during remote teaching have been recently reported solely with smaller courses [7, 27], sometimes providing different time slots for students, thus significantly increasing the instructor’s workload [8].

As noted above, apart from anonymous participation via the classroom response system, interaction between students and the instructor was rather sparse and similar to that of face-to-face courses. Inquiries via chat in synchronous lectures were often related to organizational rather than content issues, and cameras were turned off. This was the case even though students expressed that they preferred synchronous videoconference lectures to pre-recordings to allow for live interaction, such as asking questions for clarification.

Pedagogical challenges of face-to-face instruction with interaction seem to be the same or are emphasized in online instruction, e.g. interaction with and support of individual students by instructors in large courses. In any case, both students and lecturers need to take conscious advantage of opportunities and offerings. Moreover, videos and lectures should be considered in the context of the overall course curriculum, as all forms of learning materials and employment opportunities provided work in tandem for student success [10].

### Videos to complement lectures

We expect a general and lasting return to in-person teaching sooner rather than later (but may stand corrected in due course). Although some lecturers appear to have already concluded that the online experience will not influence their teaching later on [6], at this point in time it is unclear what impact the recent adaption of remote teaching may have. Some may have been surprised by the unexpected possibilities, such as the general suitability to teaching and acceptance by students. However, technical difficulties, missing interaction with students, or institutional support may have repelled other lecturers. Nevertheless, it can be reasonably assumed that lecture videos and their recordings have been so widely utilized that they will be on the minds of lecturers when planning courses in the foreseeable future. In addition, it can be expected that either segments of the student body or institutions will push to make face-to-face recordings available to students who cannot attend lectures in person. This must be carefully weighed in light of the lecturers’ performance rights and the rights of the students in attendance, as they too may be recorded. The latter, in particular, can then pose a significant barrier to extended interactions between instructors and students. While these intrusions may be tolerable in exceptional circumstances, they may not be in the long run. Moreover, the use of third-party material in the course of teaching is commonly protected, which is not the case with the public release of lecture recordings. Hence, access to lecture recordings need to be limited to participants of a particular course, i.e. they may not even be shared within an institution.

**Fig. 2:**
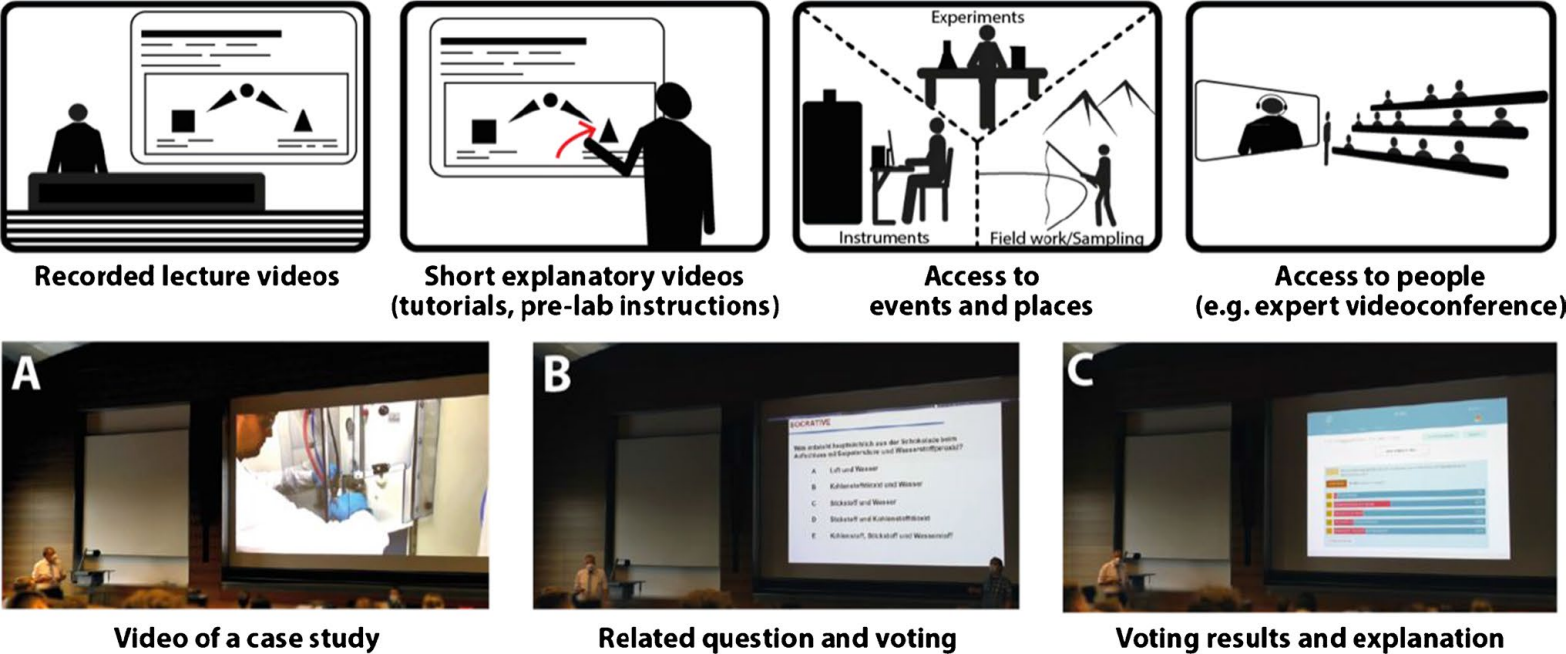
Top: Potential uses of videos to complement lectures. Bottom: Possible sequence of using a video during a lecture to introduce quantitative analysis. (**A**) A video (ca. 8 min) illustrates with processes in and outside the lab a case study (cadmium determination in chocolate). The video is commented live by the lecturer. (**B**) A related question is put to the students (multiple-choice question via a classroom response system related to chocolate sample digestion). (**C**) The vote distribution is displayed and the solution explained. Alternatively, students may be asked for a prediction for an experimental result before a video is shown

On the other hand, videos can play a role in supplementing lectures rather than taking over entire lectures or parts of them (Fig. 2, top). While videos do not provide hands-on experience [33], they can make locations and processes within a lecture hall accessible. Particularly in teaching analytical chemistry, it is often impractical to bring either the instruments to the students or all the students to the instruments when they are being explained. Similarly, some procedures may take much longer or take place in a remote location: field sampling, sample preparation, repeated measurements, etc.

Over the past few years, we have gathered some experience with short, in-house-produced videos that we use in our courses. For example, we show a video about the determination of cadmium in chocolate, starting with the motivation and underlying question and ending with the final result, introducing students to quantitative element analysis in their first lecture (Fig. 2, bottom). These videos have also been useful during distance learning. Although they require considerable effort (e.g. 3 days to plan, record, and edit the 8-min chocolate video), it is not unreasonable to produce a video each year that fits a particular course, thus building up a stockpile of videos to support the course. Since many faculty already use some type of presentation software, incorporating video clips is often simple and straightforward. A potentially more attractive option in terms of sensational impact and using recently gained experience is to livestream an experiment from the lab to the lecture hall.

As another example, we used a supplementary video clip from Ebert et al. [34] featuring the introduction of individual droplets into an inductively coupled plasma in conjunction with a previously described quiz question (question 2 in Box 1 in [24]). This does not follow the “show, don’t tell” storytelling technique, but advocates for “show, elaborate, and engage students in a reasonable manner with it”. We would like to refer interested readers to the extensive list of videos related to analytical techniques and instrumentation provided as Supplementary Information in Garza et al. [33].

## Conclusions

First of all, despite technological advances, adequate staff resources for distance education remain critical to support both faculty and students. At our institution, there were many dedicated teaching assistants and technical support staff who were able and willing to help with the sudden transition to remote teaching and solve any problems that arose.

We used both pre-recorded lectures and synchronous videoconferencing for our course. Both of these forms were helpful to students and meaningful for learning. Similar to large-enrolment courses in a lecture hall, these are not ideal but can also be effective. Students preferred synchronous lectures to stay motivated and have the opportunity to ask the instructor questions directly, but rarely used the latter option. Unlike lab courses, the rapid transition from face-to-face lectures to an online format is less demanding to meet instructional objectives. The offline lecture recording without adapting to the format may result in an unengaging product, which lacks not only the human side of the videoconference lecture but also the entertaining character of professional video productions. Following the pandemic, this may require more attention and often technical and financial support as well as appreciation from institutions.

Moreover, despite recent experience with video lectures, there are opportunities for videos to complement lectures, thereby augmenting instruction beyond simply recording presentations, which includes access to tools, locations, and procedures.

## Supplementary Information

Below is the link to the electronic supplementary material.

Supplementary file1 (PDF 622 KB)

## Footnotes

1 This is not an entirely new development as lectures have been recorded and broadcasted for decades. As an anachronistic example, you can find an advertisement in a 1973 issue of Science magazine to purchase tape recordings of meetings of the American Chemical Society for $12.50 [*Science*, **1973**, *180*, 362].

2 Note that this surveyed the entire course Analytical Chemistry II, i.e. including NMR and separation methods.
