# Supplementary material for: On video lectures during remote teaching and beyond
Source: Anal Bioanal Chem. 2022 Mar 19;414(11):3301–9. doi: 10.1007/s00216-022-03983-y (PMC8933060; doi:10.1007/s00216-022-03983-y)
Supplement: Supplementary file 1 — Supplementary file1 (PDF 622 KB) [file 216_2022_3983_MOESM1_ESM.pdf]

Supplementary Information

On video lectures during remote teaching and beyond

Gunnar Schwarz[1,*], Davide Bleiner[2, 3], and Detlef Günther[1,*]

[1] Laboratory of Inorganic Chemistry, Department of Chemistry and Applied Biosciences, ETH Zurich, Vladimir-Prelog-Weg 1, 8093 Zürich, Switzerland.

[2] Laboratory for Advanced Analytical Technologies, Swiss Federal Laboratories for Materials Science & Technology (Empa), Überlandstrasse 129, 8600 Dübendorf, Switzerland.

[3] Department of Chemistry, University of Zurich, Winterthurerstrasse 190, 8057 Zürich, Switzerland.

* Correspondence to: schwarzg@ethz.ch, detlef.guenther@sl.ethz.ch

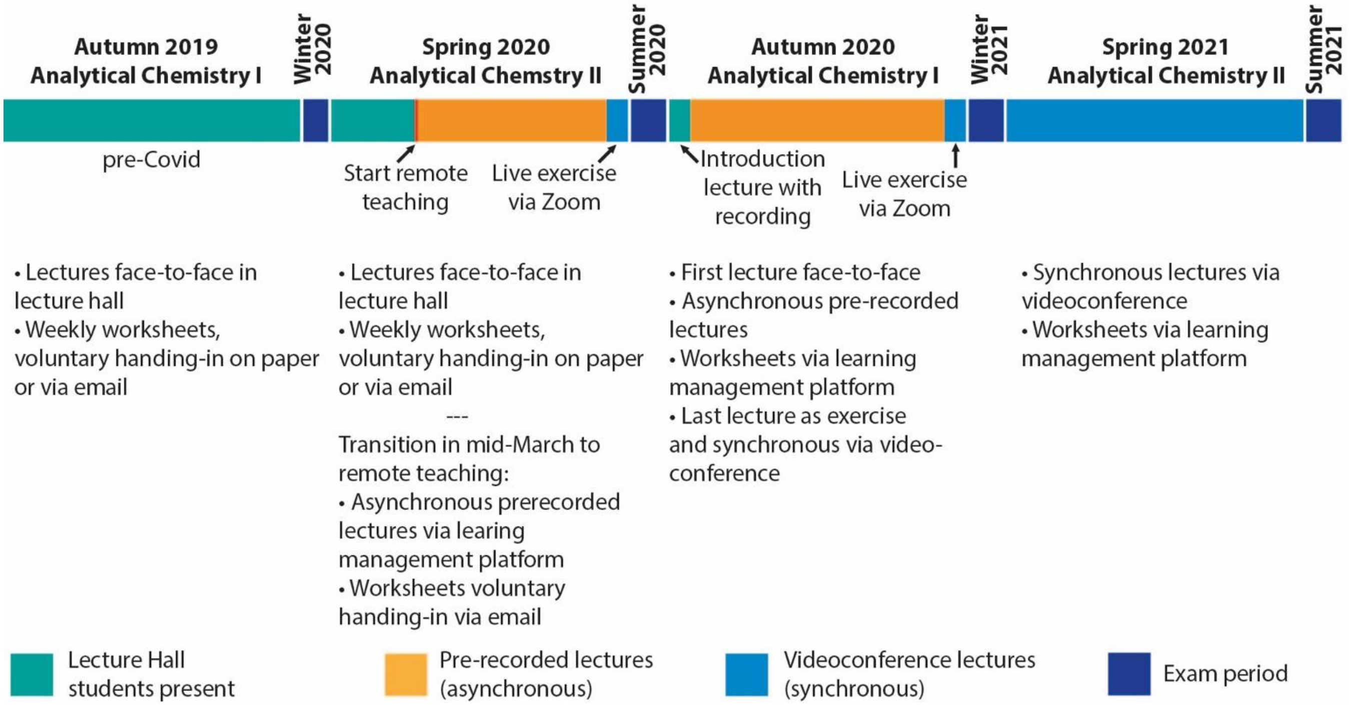

Figure S1. Timeline and lecture modalities.

Asynchronous lecture videos

The recordings took place well in advance to the scheduled time in a large lecture hall with ca. 180 seats. The lectures used presentation software and projection of slides onto the large screen (cf. [1]). In addition, technical equipment for audio and video recording was utilized, consisting of a high definition video camera (HC-V777,

Panasonic) and a wireless microphone system (Wireless Go and Lavalier Go, Røde). The camera was placed on a tripod in the middle of the lecture hall, about 10 m from the lecturer capturing both the lecturer and the presentation screen. Besides the lecturer only a teaching assistant was present during the recordings, seated next to the camera. The distance enabled the lecturer to present the material without a mask. The teaching assistant monitored the recordings. Critically, the audio signal was monitored with a headphone set plugged into the camera. This enabled the assistant to figure out and immediately intervene during a few instances, e.g. the microphone was continuously scratching along the lecturer's shirt.

Following the recordings, for each lecture a video was edited using iMovie (Apple Inc., currently version 10.1.14). Editing involved:

- adding ETH Zurich corporate design video openings and closings (in total 6 s per video),
- adding a lecture title,
- cutting pauses,
- adding black background and with an overlay of text for the polling questions (4 s), generating a short countdown (3 s) until the lecture resumes,
- as required, overlay of a specific presentation slide for improved visibility,
- as required, overlay of a video file, which would have been otherwise displayed in the lecture hall on the presentation screen, and
- as required, white balance correction using white space from a presentation slide.

The video was first exported from iMovie in high quality, 720p or 1080p, and "faster" compression. To reduce the file size further, each video file was compressed using an open source video transcoder (Handbrake, handbrake.fr), so that a video file was less than 500 MB per 30 min lecture at 1080p. In hindsight, higher compression would also be suitable, if no rapid movement or fine details (e.g. on the slides) need to be discernible. Editing and video compression required less than 10 min of workload without considering computation and one hour in total per lecture video.

Note that video recordings should be tested, in particular whether the audio and video quality is sufficient for the intended purpose. Moreover, if any projection of slides via beamers is used, aliasing is possible due to the projection and sampling frequencies of the camera resulting in irritating artefacts in the final video, i.e. moving lines across the projection screen.

The videos were made available from the time of the scheduled lectures via a learning management system (Moodle). Presentation slides were made available at least a day in advance of the lecture, but leaving out slides containing tasks or questions.

We included prompts for classroom response system polls into the videos and used it as described elsewhere[1]. The polls for a lecture were open during the entire day, but only a few responses were received after the scheduled lecture time slot. In the final videos, the question and option for multiple-choice questions are presented, followed by an intermediate overlay (ca. 4 s) with an explicit prompt to stop the video, think about the question, and respond via the classroom response system. Then a short countdown (3 s) signalled the continuation of the lecture with the solution and brief explanation. As with face-to-face lectures, the questions,

vote distributions, solutions and explanations were published a little later in the learning management system in a separate document. Hence, students were not able to view live polling results in asynchronous video lectures.
To maintain accessible to students for any inquiries, we held virtual office hours at the end of the lecture time slot and encouraged students to contact us via email and leave comments/feedback via the classroom response system (anonymously) or learning management system at the end of the online problem sets.

Synchronous videoconference lectures

The synchronous lectures were held using the videoconference provider Zoom (zoom.us) with accounts provided by the institution during the allocated time slots and a single meeting ID. Students were free to set the display layout (positioning of presentation slides and lecturer video stream) individually as far as the software allowed, but for the recordings (managed by the lecture assistant) a layout shown in Figure 1 was maintained.
The recording were subsequently and slightly edited (adding ETH Zurich corporate design video openings and closings, clipping video edges before and after the actual lectures, cuts of some waiting times (e.g. during polls), and as the case maybe add black overlays to a few sections when students' video feeds were visible to protect their privacy as their homes were on display). This took less than 10 min per lecture video. The final videos were made available via the learning management system.

Reference

1. Schwarz, G., *Interface Model and Implementation Framework for Classroom Response Systems.* Journal of Chemical Education, 2021. **98**(6): p. 2122-2127.
